# Cerato-platanin family proteins: one function for multiple biological roles?

**DOI:** 10.3389/fpls.2014.00769

**Published:** 2015-01-06

**Authors:** Ivan Baccelli

**Affiliations:** Dipartimento di Scienze delle Produzioni Agroalimentari e dell'Ambiente, Università degli Studi di FirenzeFirenze, Italy

**Keywords:** cell wall loosening, fungal growth, turgor pressure, virulence, MAMP, effectors, expansins, snodprot

## Introduction

Despite the increasing number of sequenced genomes, cerato-platanin family proteins (CPPs) seem to exist only in fungi. Bacteria, oomycota, plants, and animals do not in fact possess CP homologs, whereas genes codifying for CPPs have been found, so far, in more than 50 fungal genomes (Chen et al., 2013).

Among these fungi there are plant pathogens, such as *Botrytis cinerea* and *Magnaporthe grisea* (Ascomycota), *Heterobasidion irregulare* and *Moniliophthora perniciosa* (Basidiomycota), but also biocontrol agents (e.g., *Trichoderma* spp.), mycorrhizal fungi, saprotrophs, and human pathogens.

Experimental data suggest that CPPs play a role during fungus-plant interactions. When *T. virens* and *T. harzianum* were respectively co-cultured with cotton or tomato roots, the expression of CPPs increased compared to the culture obtained in the absence of the plant (Djonović et al., 2006; Samolski et al., 2009); in *B. cinerea* and *M. grisea*, knockout mutants for *CPP* genes showed reduced virulence on their plant hosts (see Table 1) (Jeong et al., 2007; Frías et al., 2011).

**Table 1:** **Summary table of the mutants obtained up to date for genes encoding cerato-platanin family proteins (CPPs)**.

**Fungi mutated for *CPP* genes**	**Phenotypic analysis**	**References**	**Expansin-like genes in the genome**
*Trichoderma virens*	Neither deletion nor overexpression of *sm1* affected normal growth or development of *T. virens*, conidial germination, production of gliotoxin, hyphal coiling, hydrophobicity, or the ability to colonize maize roots	Djonović et al., 2007	Yes
*T. atroviride*	Single and double knockout strains for *epl1* and *epl2* did not differ in the growth properties and biomass formation *in vitro*, formation of aerial hyphae, bridging of gaps between two agar blocks, transition of hyphae between solid/liquid interfaces, osmotic, desiccation and cell wall stresses, conidiation, germination efficiency, hydrophobicity of the mycelium, chlamydospore formation, and mycoparasitic ability	Frischmann et al., 2013	Yes
*Magnaporthe grisea*	*msp1* mutants were phenotypically indistinguishable from wild type and elaborated apparently normal appressoria. However, the deletion mutants were greatly reduced in virulence on rice and barley primarily due to impaired growth in planta	Jeong et al., 2007	Yes
*Botrytis cinerea*	*bcspl1* mutant strains showed reduced virulence in a variety of hosts, such as tomato and tobacco plants, and some fruits. Growth rates on solid media, conidia production, and wettability of the fungal colonies were similar to the wild type	Frías et al., 2011	Yes
*Leptosphaeria maculans*	*sp1* was not crucial for pathogenicity on *Brassica napus* cotyledons	Wilson et al., 2002	Yes

Plants have developed the ability to recognize CPPs and to activate defense responses when in contact with them. In fact, CPPs have been reported to act as microbe/pathogen-associated molecular patterns (MAMPs/PAMPs) (for reviews, see Gaderer et al., 2014; Pazzagli et al., 2014).

The secretion of these small proteins (120–130 a.a.) seems to be universal in fungi. CPPs are often the most present in the culture filtrates, but in some studies they have been found localized in the fungal cell walls (Boddi et al., 2004; Seidl et al., 2006; Shah et al., 2009; Frías et al., 2014).

Gene knockout experiments have not yet permitted the identification of a clear biological function, and to date it is not easy to answer the question of why fungi produce CPPs. Gene expression data and studies on their biochemical properties have led to the formulation of various hypotheses that may appear different for each homolog. In summary, it is generally believed that CPPs play at least two roles in fungi: one in growth and development (generally referred to as the primary role), which should justify their presence in the cell wall, and one, more elusive, that should explain their secretion and the interaction with plants (Gaderer et al., 2014; Pazzagli et al., 2014). But what is their function?

This opinion article will attempt to answer these questions: can we hypothesize a basal function which characterizes CPPs and allows them to have multiple biological roles depending on the context (fungal cell wall or extracellular environment)? Can this function imply a role in plant colonization?

## Cerato-platanin family proteins and expansins: more than a structural similarity

CPPs are not hydrophobin-like proteins as initially hypothesized, because they have different biochemical and structural properties (Frischmann et al., 2013). On the contrary, they are similar to expansins for many aspects.

Structurally, CPPs have a single domain (named “cerato-platanin domain”) which forms a double Ψβ-barrel fold similar to the domain 1 (D1) of expansins (de Oliveira et al., 2011; de O Barsottini et al., 2013). Expansins contain two domains: the N-terminal domain (D1) forms the double Ψβ-barrel fold; the C-terminal domain (D2) has an immunoglobulin-like β-sandwich fold and is similar to a family of grass-pollen allergens (Yennawar et al., 2006; Georgelis et al., 2011).

Expansins and expansin-like proteins are possessed by plants, fungi, bacteria, and other organisms (Nikolaidis et al., 2014). In plants, expansins mediate the turgor-driven extension of plant cell walls, and they play multiple roles in plant physiology and development, including growth, fruit softening, organ abscission, and pollen tube penetration of the stigma (Sampedro and Cosgrove, 2005). In microbes, their role is still elusive (Georgelis et al., 2014).

To date, no enzymatic activity has been found for expansins, and their molecular mechanism of action has not yet well-defined. The idea is that they break non-covalent interactions between cell wall components, allowing polymer slippage (creep), and cell wall loosening (Yennawar et al., 2006).

Since expansins can weaken cellulosic substrates, their possible use in biofuel production as enhancers of enzymatic hydrolysis has received great interest (Arantes and Saddler, 2010). Like expansins, CPPs did not show any enzymatic activity but were able to weaken cellulosic materials (de O Barsottini et al., 2013; Baccelli et al., 2014).

There are other interesting similarities between CPPs and expansin-like proteins that are worth summarizing: (1) CPPs, as well as expansins, have been found localized in the cell walls (Sampedro and Cosgrove, 2005; Bouzarelou et al., 2008; Gaderer et al., 2014); (2) gene expression data link both CPPs and expansins with growth and developmental processes (including, for CPPs, the formation of spores and conidia) (Cosgrove et al., 2002; Gaderer et al., 2014); (3) CPPs and fungal expansins can bind chitin (Chen et al., 2010; Baccelli et al., 2014; Gaderer et al., 2014); (4) the optimum of activity of these proteins usually occurs at an acid pH (Sampedro and Cosgrove, 2005; Chen et al., 2010; Baccelli et al., 2014); (5) gene knockout experiments have often led to phenotypes that are identical to wild types (for CPPs, see Table 1) (Cosgrove et al., 2002; Bouzarelou et al., 2008).

Conversely, CPPs have a main difference compared to expansins: they do not bind to cellulose, a property that seems to be universal in plant and microbial expansins (Yennawar et al., 2006; Georgelis et al., 2011; Gaderer et al., 2014).

In view of the similarities between CPPs and expansins, it seems possible to suppose that CPPs, when localized in the fungal cell wall, can act by disrupting non-covalent interactions between fungal cell wall components: for example, between β-glucan or chitin chains. This function could explain their localization and gene regulation. In fact, they could act in hyphal elongation, in the formation of spores and fruiting bodies, spore germination, and in all those processes requiring remodeling and enlargement of the fungal cell wall. It is worth reporting that the cerato-platanin MpCP2 was able to facilitate the germination of basidiospores in *M. perniciosa* (de O Barsottini et al., 2013). The idea is that this ability to act on non-covalent bonds is due to the fold that CPPs and expansins both have. Moreover, CPPs and expansins have a conserved aspartate within the shared domain (Asp-77 and Asp-82, respectively), which is crucial in expansins for the wall extension activity (Georgelis et al., 2011).

Interestingly, in a recent study concerning the elongation growth of the stipe of* Flammulina velutipes*, it has been found that a protein from the snail *Helix aspersa* was able to induce stipe wall extension without hydrolytic activity (Fang et al., 2014). Although this 21-kDa protein was not sequenced, this paper has reported for the first time the ability of an expansin-like protein to extend a cellulose-free wall. Interestingly, the cell wall extension was measured with an extensometer in a test analogous to that used for plant expansins. In conclusion, the hypothesis is that CPPs can act on non-covalent interactions both in cellulose- and chitin-containing walls.

If CPPs have this important function in the fungal cell wall, how can we explain that gene knockout strains did not show any phenotype related to hyphal growth or development?

As shown in Table 1, all mutated fungi possess both CPPs and expansin-like proteins in their genomes. In *Trichoderma* these proteins are called swollenins (Brotman et al., 2008). In other fungi, they may be called loosenins (Quiroz-Castañeda et al., 2011). Multiple knockout strains for CPP and expansin-like genes have never been obtained, we can therefore suppose that it is necessary to produce these multiple mutants to avoid compensation effects in fungi.

## Role in plant colonization: cerato-platanin proteins may facilitate the mechanical penetration of fungi

Not all CPPs are localized in the fungal cell wall (Jeong et al., 2007), whereas all CPPs are abundantly secreted outside the hyphae during growth. When knockout mutants were obtained, they never revealed differences in morphological traits compared to wild types; however, some studies have reported a reduced virulence of the mutants when tested on host plants (Table 1). This first of all implies that CPPs must have a role in the interaction with plants, as already hypothesized (Pazzagli et al., 2014); but how can they promote the interaction? Above all, can their function be useful to non-pathogenic fungi like *Trichoderma*?

Recently, it has been reported that MpCP5, a CPP from *M. perniciosa*, when pre-incubated with N-acetylglucosamine oligomers bound to them and prevented the defense responses of tobacco seedlings (de O Barsottini et al., 2013). This could actually be a role for CPPs during the interaction with plants, i.e., as scavengers of chitin fragments produced by plant chitinases, but we do not know whether during the invasion process this property can be effective, because a direct competition assay between the scavenger and the plant receptor should be performed.

Pathogenic fungi invade their hosts both through the secretion of extracellular lytic enzymes and by exerting a physical force on the tissues (Bellincampi et al., 2014). The force is provided by the enormous turgor pressure that fungi are able to generate. *M. grisea* generates the turgor pressure within the appressoria by increasing the intracellular glycerol, and the pressure is applied as a physical force able to break the leaf cuticle (Bastmeyer et al., 2002; Serrano et al., 2014).

Recently, three CPPs from different fungi (CP from *Ceratocystis platani*, Pop1 from *C. populicola* and MpCP2 from *M. perniciosa*) have been reported to weaken filter paper without any hydrolytic activity (de O Barsottini et al., 2013; Baccelli et al., 2014). Cellulose fibers were loosened and dispersed after incubation with the protein under shaking, which actually is an external force applied to the incubation mixture. As CPPs were not tested with an extensometer, it is not possible to compare their activity with that of expansins. For example, the incubation of paper strips with the bacterial expansin EXLX1 reduced the breakage force by 20% (Georgelis et al., 2011). Therefore, this experiment and some tests on plant cell walls should be performed in future.

CPPs, through their demonstrated non-enzymatic activity on cellulose (or other plant cell wall components that should be tested), could reduce the force needed to break the plant cell walls and thus facilitate the hyphae during the mechanical penetration.

*Trichoderma* is able to penetrate the root tissues and colonize several epidermal layers (Brotman et al., 2008). The function here delineated would thus be useful to non-pathogenic fungi like *Trichoderma*, and also to mycorrhizal fungi during the establishment of the contact surface (Balestrini and Bonfante, 2014).

We cannot exclude that also fungal expansins, when secreted, play a similar role. However, in that case we can assume that CPPs are more functional: this could indeed explain why some deletion mutants would not be blurred by the presence of expansin-like proteins in their genomes. It is plausible that CPPs, because of their inability to bind cellulose, are more functional than expansins. This property would allow fungi to have proteins always active around the hyphae, and to reduce their dispersal during the penetration of the plant cell walls. Moreover, CPPs could be kept near the fungus thanks to their chitin-binding ability.

The invading strategy and the balance between the secretion of lytic enzymes and the use of mechanical pressure could, in any case, influence the importance of CPPs in each fungus-plant interaction.

## Concluding remarks

CPPs are well-known to act as elicitors on plants and seem to possess all the requirements to be classified as MAMPs, because they are widespread in fungi, have a conserved structure, are present both in pathogenic and non-pathogenic fungi, and are abundantly secreted. But what is their function in fungi? On the basis of recent results, this Opinion Article has tried to suggest the answer.

The idea is that CPPs can act in an expansin-like manner on non-covalent interactions and cause the loosening of fungal and plant cell walls. This basal function could explain why CPPs have multiple biological roles in fungal life. At the same time, CPPs and expansin-like proteins might have similar functions in fungi, and a few gene knockout experiments could have been blurred by expansins. In the host colonization, CPPs would act as facilitators of the mechanical penetration, and their ability to act on cellulose without binding to it would make them very functional.

### Conflict of interest statement

The author declares that the research was conducted in the absence of any commercial or financial relationships that could be construed as a potential conflict of interest.
